# Glucocerebrosidase and its relevance to Parkinson disease

**DOI:** 10.1186/s13024-019-0336-2

**Published:** 2019-08-29

**Authors:** Jenny Do, Cindy McKinney, Pankaj Sharma, Ellen Sidransky

**Affiliations:** Section on Molecular Neurogenetics, Medical Genetics Branch, National Human Genome Research Institute, National Institutes of Health, Building 35A, Room 1E623, 35 Convent Drive, MSC 3708, Bethesda, MD 20892-3708 USA

**Keywords:** Gaucher disease, *GBA1*, Parkinson disease, Glucocerebrosidase, α-Synuclein, Lysosome

## Abstract

Mutations in *GBA1*, the gene encoding the lysosomal enzyme glucocerebrosidase, are among the most common known genetic risk factors for the development of Parkinson disease and related synucleinopathies. A great deal is known about *GBA1*, as mutations in *GBA1* are causal for the rare autosomal storage disorder Gaucher disease. Over the past decades, significant progress has been made in understanding the genetics and cell biology of glucocerebrosidase. A least 495 different mutations, found throughout the 11 exons of the gene are reported, including both common and rare variants. Mutations in *GBA1* may lead to degradation of the protein, disruptions in lysosomal targeting and diminished performance of the enzyme in the lysosome.

Gaucher disease is phenotypically diverse and has both neuronopathic and non-neuronopathic forms. Both patients with Gaucher disease and heterozygous carriers are at increased risk of developing Parkinson disease and Dementia with Lewy Bodies, although our understanding of the mechanism for this association remains incomplete. There appears to be an inverse relationship between glucocerebrosidase and α-synuclein levels, and even patients with sporadic Parkinson disease have decreased glucocerebrosidase. Glucocerebrosidase may interact with α-synuclein to maintain basic cellular functions, or impaired glucocerebrosidase could contribute to Parkinson pathogenesis by disrupting lysosomal homeostasis, enhancing endoplasmic reticulum stress or contributing to mitochondrial impairment. However, the majority of patients with *GBA1* mutations never develop parkinsonism, so clearly other risk factors play a role. Treatments for Gaucher disease have been developed that increase visceral glucocerebrosidase levels and decrease lipid storage, although they have yet to properly address the neurological defects associated with impaired glucocerebrosidase. Mouse and induced pluripotent stem cell derived models have improved our understanding of glucocerebrosidase function and the consequences of its deficiency. These models have been used to test novel therapies including chaperone proteins, histone deacetylase inhibitors, and gene therapy approaches that enhance glucocerebrosidase levels and could prove efficacious in the treatment of forms of parkinsonism. Consequently, this rare monogenic disorder, Gaucher disease, provides unique insights directly applicable to our understanding and treatment of Parkinson disease, a common and complex neurodegenerative disorder.

## Background

Of all the known genetic variants associated with Parkinson disease, mutations in *GBA1*, the gene encoding the lysosomal enzyme glucocerebrosidase (Glucosylceramidase Beta or GCase; EC 3.2.1.45), have a major advantage due to the association of this gene with a well-studied lysosomal storage disorder, Gaucher disease. Gaucher disease, an autosomal recessively inherited disorder with diverse clinical manifestations, was first described in Paris over 135 years ago by a medical student, Philippe Gaucher, who examined a patient with a massively enlarged spleen [1]. It was not until a half century later that it was discovered that the stored material found in patients with this disorder was in fact a glycolipid, glucosylceramide (GlcCer) [2]. In 1965, Dr. Roscoe Brady at the National Institutes of Health in Bethesda, Maryland determined that Gaucher disease resulted from an enzymatic defect in the lysosomal enzyme glucocerebrosidase (GCase), that normally cleaves a glucose moiety from GlcCer [1, 3]. This finding facilitated purification of the protein GCase, the cloning of the *GBA1* gene in 1981, and the development of enzyme replacement therapy (ERT) as a treatment for patients with Gaucher disease [4]. Indeed, much work in the past decades has focused on mutations in *GBA1* and their phenotypic consequences. Thus, unlike other newly discovered Parkinson genes, a great deal is known about *GBA1* and the function of its resulting enzyme, GCase.

### Glucocerebrosidase: biochemistry and molecular biology

**Fig. 1:**
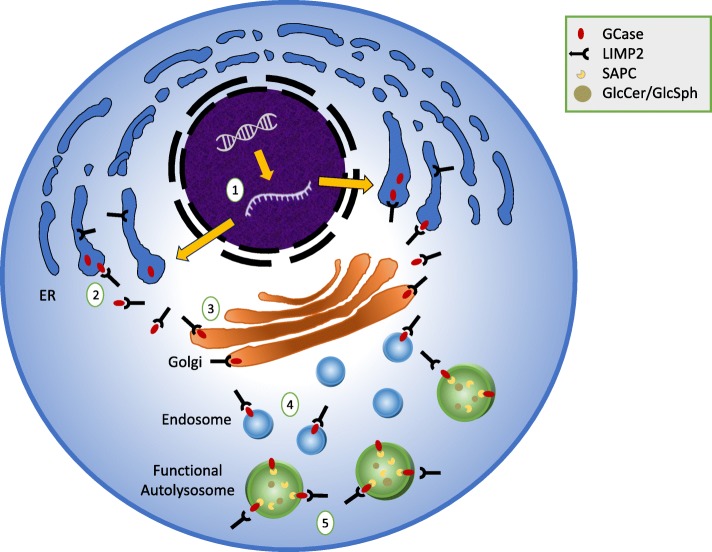
Simplified diagram of the synthesis and trafficking of GCase in a functional cell. 1) *GBA1*, the gene coding for GCase, is transcribed into mRNA that is then transported out of the nucleus to the ER. 2) GCase is synthesized in the ER, where it binds to the protein LIMP2 in the favorable neutral pH of the cytoplasm. 3) LIMP2 transfers GCase through the Golgi. 4) GCase is then transferred to a late endosome. 5) When the late endosome fuses with a lysosome to form an autolysosome, LIMP2 disengages from GCase due to the decrease in pH. In the lysosome, GCase is activated by SAPC. GCase actively hydrolyzes its substrates GlcCer and GlcSph in this compartment

**Fig. 2:**
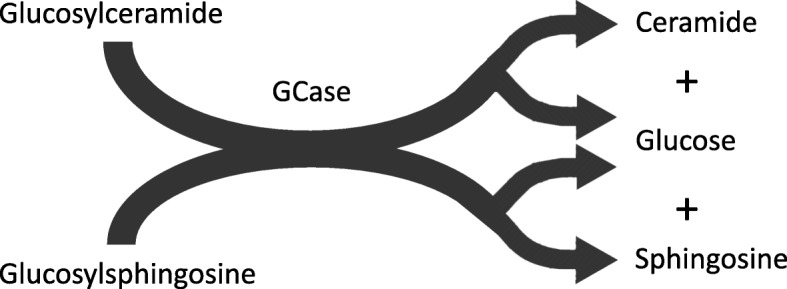
Reaction schema depicting the enzyme GCase hydrolyzing GlcCer and GlcSph. In the lysosome, GCase hydrolyzes substrates GlcCer (above) and GlcSph (below) by cleaving a glucose moiety off the molecule, creating the products glucose and ceramide, or glucose and sphingosine, respectively

GCase is a 497-amino-acid membrane-associated protein with a 39-amino-acid leader sequence and five glycosylation sites [4, 5]. The protein is synthesized in the endoplasmic reticulum (ER) and glycosylated, but the enzyme only becomes active when transferred to the acidic lumen of the lysosome (Fig. 1). Unlike other lysosomal proteins that are targeted to the lysosome by mannose-6-phosphate receptor dependent pathways, GCase is transported from the ER by the GCase transporter lysosomal integral membrane protein-2 (LIMP2), encoded by the gene *SCARB2* [6]. Once in the lysosome, the enzyme interacts with another partner, its activator protein saposin C (SAPC) [7], a subunit of the precursor protein, prosaposin (PSAP). In the lysosomal compartment, the enzyme hydrolyzes glucose moieties from both GlcCer and glucosylsphingosine (GlcSph) (Fig. 2).

**Fig. 3:**
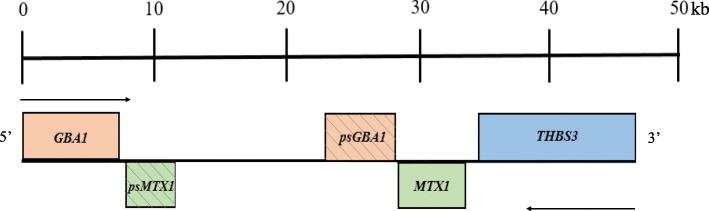
Scaled map of a 50 kb gene-rich region surrounding/antecedent to the *GBA1* gene on chromosome 1q21. Genes represented above the line are transcribed right to left, while the genes below are transcribed left to right. Note the close proximity of *GBA1* to its pseudogene with 98% homology, making it a common site for recombination events [9]

The *GBA1* gene is located in a gene-rich region on chromosome 1q21. It is composed of 11 exons and includes around 7000 base pairs of DNA [8]. A highly homogenous, untranslated pseudogene that shares 98% homology in the coding regions is located only 16 kb downstream. A second gene, metaxin 1 (*MTX1*), encoding a protein located in the outer mitochondrial membrane, is located downstream to the *GBA1* pseudogene sequence and is convergently transcribed [9]. There is also a *MTX1* pseudogene located in between *GBA1* and its pseudogene. The gene for thrombospondin 3 (*TPS3*), a glycoprotein that mediates cell-to -matrix and cell-cell interactions, is immediately downstream to *MTX1* (Fig. 3).

At least 495 known *GBA1* mutations are associated with Gaucher disease, the majority being missense mutations [10, 11]. Mutation nomenclature is complicated, as the numbering of the mutated amino acid was changed several years ago to include the 39-amino-acid leader sequence (newer numbering shown in parentheses). There are two common mutations found in patients. The mutation N370S (p.N409S), found exclusively in patients with type 1 Gaucher disease, is the most frequent mutation encountered among patients in the United States, Europe and Israel. The L444P (p.L483P) mutation is found world-wide, and when homozygous, is frequently associated with neuronopathic Gaucher disease. Other identified mutations, located across all exons of *GBA1*, include point mutations, frame shifts, splicing mutations, and null alleles often resulting from recombination with the homologous pseudogene sequence [12]. Many *GBA1* mutations are relatively common, but others are rarer, found only in individual families.

### Gaucher disease

Overall, Gaucher disease is a pan-ethnic disorder affecting between 1 in 50,000 to 1 in 100,000 people world-wide [4]. The frequency of Gaucher disease is increased in the Ashkenazi Jewish population where the carrier frequency is between 1 in 14 to 1 in 18. Screening for 6–8 specific *GBA1* mutations can identify around 95% of mutant alleles in Ashkenazi Jewish patients with Gaucher disease, while the genotypic diversity is far broader in other ethnicities.

Gaucher disease results from the deficiency of lysosomal GCase and the accumulation of the lipid substrates GlcCer and GlcSph within the lysosomes of macrophages. These engorged cells are referred to as “Gaucher cells” and have a unique “crumpled tissue paper-like” appearance on hematoxylin and eosin staining. Electron microscopy of Gaucher macrophages show inclusions with a tubular structure [13]. These distinctive cells are commonly found in the spleen, liver, lung and bone marrow, leading to symptoms in these specific organs. Painless splenomegaly is often the first sign of Gaucher disease and is sometimes accompanied by hepatomegaly. Thrombocytopenia and anemia are also quite common. Bone disease, including painful bone “crises”, as well as fractures and osteopenia, are significant causes of morbidity in patients.

By definition, patients who have no neurological involvement as a result of their GCase deficiency are considered to have type 1 or non-neuronopathic Gaucher disease. Among patients with type 1 Gaucher disease, symptoms are highly variable, and the disease can present at any age. Phenotypes include children with cytopenia and organomegaly, adolescents with bone pain and fractures requiring orthopedic surgery, and asymptomatic older adults. While numerous mutations have been identified in patients with type 1 Gaucher disease, mutation N370S is the mutation most commonly encountered, and homozygosity for N370S is often seen among the patients with the mildest phenotypes. However, genotype-phenotype associations have limited value, as even some patients with genotype N370S/N370S develop serious disease complications [14].

Brain involvement resulting from GCase deficiency can also occur, and the associated manifestations are quite diverse. Acute neuronopathic or type 2 Gaucher disease presents perinatally or in the first months of life [15]. It is a devastating disorder accompanied with neurodegeneration and brainstem involvement. Infants have organomegaly, failure-to-thrive, and compromised swallow and airway problems. Associated genotypes include homozygosity for a recombinant allele, frame-shift mutation, or other severe mutations. Compound heteroallelic genotypes comprised of functionally “null” mutations together with a L444P allele are also associated with type 2 Gaucher disease [16].

Patients with any neurologic involvement who do not fit into the category of type 2 Gaucher disease are considered to have type 3 Gaucher disease, which is a very phenotypically diverse group. The most common and perhaps defining manifestation encountered in patients with type 3 Gaucher disease is the slowing or looping of the horizontal saccadic eye movements. Other features described include myoclonic epilepsy, generalized seizures, and learning and behavioral difficulties. However, in some patients, the eye movement findings are the sole neurologic manifestation [17].

Non-neuronopathic Gaucher disease and the visceral manifestations of type 3 Gaucher disease can be effectively treated. Enzyme replacement therapy (ERT), available since 1991, consists of biweekly intravenous infusions of recombinant GCase [18]. Substrate reduction therapy (SRT), an oral drug inhibiting the synthesis of GlcCer, is available for adults with Gaucher disease and is also shown to reverse visceral disease manifestations [19]. Nevertheless, ERT and SRT therapies are extremely costly; moreover, ERT does not cross the blood-brain-barrier, and is therefore unable to prevent neurodegeneration. While the most widely used SRT is not brain penetrant, others that can cross the blood-brain barrier are undergoing clinical trials [20]. Alternate strategies, including small molecule chaperones and gene therapy, are being explored and developed.

### *GBA1* and parkinsonism

The association between mutations in *GBA1* and the development of parkinsonism was first appreciated in the 1990’s with the identification of rare patients with Gaucher disease who also developed Parkinson disease [21–23]. It was subsequently appreciated that Parkinson disease was more common in heterozygote family members of patients with Gaucher disease [24]. Pilot studies conducted with brain bank samples [25], and in Parkinson disease clinics, suggested that patients with Parkinson disease sometimes carried *GBA1* mutations [26]. Ultimately, studies in large Parkinson disease cohorts and a multicenter international collaborative study established that in Parkinson disease world-wide, the odds ratio for a mutation in *GBA1* was greater than five [27, 28]. Today it is estimated that between 7 and 12% of patients with Parkinson disease carry a *GBA1* mutation. The frequency varies depending on the population; for example, since the carrier frequency of *GBA1* mutations is much higher among Ashkenazi Jews, over 15% of Ashkenazi Jewish patients with Parkinson disease carry at least one common *GBA1* mutation [27]. There is some indication that mutant alleles associated with more severe Gaucher disease have a higher associated risk of developing parkinsonism [29].

Despite the increased risk of developing parkinsonism among *GBA1* mutation carriers, it is important to emphasize that only a minority of carriers with *GBA1* mutations ever develop Parkinson disease. The same applies even to patients with Gaucher disease, despite exhibiting significantly attenuated GCase activity as a result of two mutated *GBA1* alleles [30]. A study from 2011 reports that in a registry of 4051 adult patients with type 1 Gaucher disease, 68 were diagnosed with Parkinson disease [31]. After age-matching, the probability of patients developing Parkinson disease was calculated as 5–7% by age 70 and 9–12% by age 80. However, these results are limited by the nature of the study design and the information available in the International Collaborative Gaucher Group (ICGG) registry, as not all Gaucher patients are registered in the ICGG, especially in cases where patients’ symptoms are so mild that their Parkinson diagnosis precedes their Gaucher diagnosis [32, 33]. Additionally, the registry does not include carriers of just one *GBA1* mutation. As a result, the incidence of Parkinson disease in patients with Gaucher disease remains difficult to quantify. However, one study noted that although the incidence of Parkinson disease is similar in homozygote and heterozygote carriers of *GBA1* mutations, the age of onset for homozygotes are approximately 6–11 years earlier than in heterozygotes [34]. Furthermore, the connection between GCase activity and Parkinson disease is complicated by the fact that two mild *GBA1* alterations that do not in themselves cause Gaucher disease, E326K (p.E365K) and T369 M (p.T408 M), still predispose patients to parkinsonism [30, 35]. While no potential mechanism has been described for these mutations, it is possible that the combined effect of the mutation with assorted environmental or non-*GBA1* genetic factors induce a higher risk for Parkinson disease. This suggests that the factors leading to Parkinson pathogenesis are multifaceted in nature and are not completely explained by deficient lysosomal activity and the accumulation of substrate. Therefore, unlike certain genes leading to familial, monogenic forms of Parkinson disease, *GBA1* mutations should be considered only as a risk factor for parkinsonism.

### The biological role of glucocerebrosidase in Parkinson pathogenesis

Since the link between Gaucher heterozygotes and Parkinson disease was established, it is recognized that deficient GCase has some biological role as a modifier or facilitator of Parkinson pathogenesis in the brain. In fact, brain autopsy studies have shown that even some cases of idiopathic Parkinson disease (without *GBA1* mutations) exhibit decreased levels of GCase [36, 37].

As mentioned, after synthesis in the ER, wildtype GCase hydrolyzes glucose from GlcCer and GlcSph in the lysosomal lumen. Once thought to be the cellular endpoint of endocytosis and removal of cellular debris, the lysosome is now recognized as a vital and interconnected organelle. It monitors nutrient status, it actively communicates with the nucleus via a master regulator, Transcription Factor EB (TFEB), it acts as a secretory center for some macromolecules, and it contains more than 60 acidic hydrolases that degrade macromolecules delivered by the endocytic and autophagic pathways [38, 39]. Several studies postulate how potential interactions in the autophagic pathway can promote Parkinson pathology, speculating that a reduction in GCase activity may enhance the risk for Parkinson disease by facilitating the pathological hallmark for Parkinson disease, α-synuclein accumulation. Many studies are now addressing how normal levels of GCase protein might interplay with α-synuclein to maintain neurological function [28, 40].

### The role of α-Synuclein in Parkinson pathogenesis

Parkinson disease, characterized by bradykinesia, rigidity, and tremor, is associated with the loss of dopaminergic neurons and by the accumulation of insoluble α-synuclein fibrils in the form of Lewy bodies and neurites in the substantia nigra of the brain. The involvement of α-synuclein in Parkinson disease was elucidated when a missense mutation (p.A53T) was documented in the α-synuclein gene (*SCNA*) in an extended Italian kindred with hereditary Parkinson disease [41, 42]. It was further substantiated when α-synuclein was found to be a component of Lewy bodies 1 year later [43]. There is some evidence suggesting that α-synuclein is a soluble protein that may exist in the cell primarily as a helical tetramer that is resistant to aggregation, although this remains controversial [44, 45]. In the cell, α-synuclein is in equilibrium between a soluble (cytosolic) form and a membrane bound form [46]. Its function remains unclear, but is speculated to be involved in exocytosis, and along with its isoforms β- and γ-synuclein, it is expressed in specific brain regions and likely involved in presynaptic neural transmissions to the dendrites of a postsynaptic neuron (reviewed in [47]). α-Synuclein requires interacting molecular partners like SNARE proteins, lipid membranes, dopamine homeostatic proteins, calcium regulating proteins, α-synuclein itself and others to function in the cell [47]. Under conditions where the local concentration of α-synuclein is high, it can self-assemble to form insoluble α-synuclein aggregates and fibrils. This aggregation process is enhanced by pre-existing aggregates, so α-synuclein can self-seed to increase these insoluble forms, much like misfolded prion proteins. α-Synuclein is cleared from the cells by macroautophagy, a general degradative cell function, and by chaperone mediated autophagy (CMA) requiring lysosome-associated membrane protein 2a (LAMP2a), another lysosomal transporter that mediates entry of molecules to the lysosome [48]. Additionally, it was shown that α-synuclein pathology reduces CMA pathway activity at its lysosomal entry receptor [49]. It is possible that the conversion of the physiologically active soluble form of α-synuclein to the insoluble aggregate form is one of many factors promoting Parkinson disease and other neurodegenerative synucleinopathies.

### The association between GCase and α-Synuclein

**Fig. 4:**
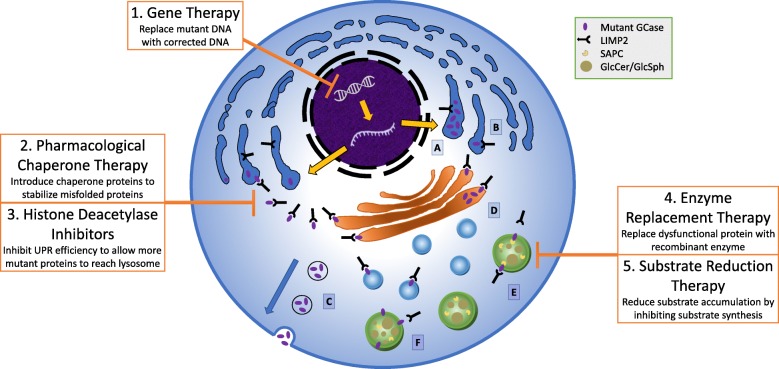
Different hypothetical mechanisms by which GCase can be impaired, and various therapeutic approaches targeting these mechanisms. These include A) failure of the GCase protein to exit the ER, B) failure of GCase to link with its LIMP2 trafficking transporter, C) GCase is misfolded and unstable, so degraded through the unfolded protein response, D) failure of GCase to exit the Golgi, E) GCase is inactive due to mutations at the active site, and F) GCase activity is altered due to a Saposin C defect, and. The failure of GCase to reach the lysosome or be activated in the lysosome enables GlcCer and GlcSph to accumulate in the lysosome, creating the hallmark marker of Gaucher disease, Gaucher cells. Various therapies to address GCase impairment include: 1) Gene therapy: directly replacing mutant DNA with corrected DNA via adeno-associated or other viral infection. 2) Pharmacological chaperone therapy: introducing chaperone proteins to stabilize and refold misfolded proteins. 3) Histone deacetylase inhibitors: inhibiting unfolded protein response to allow more misfolded proteins to reach the lysosome. 4) Enzyme replacement therapy (ERT): replacing dysfunctional enzyme with recombinant enzyme targeted to the lysosome. 5) Substrate reduction therapy (SRT): reducing substrate accumulation regardless of GCase levels by inhibiting substrate synthesis. Currently, ERT and SRT are the only FDA-approved treatment options for patients with Gaucher disease

*GBA1* mutations may structurally change the GCase protein, resulting in decreased enzymatic activity (loss of function). In theory, these consequences may occur in several ways and postulated hypotheses include: 1) failure of the GCase protein to exit the ER, 2) failure of GCase to link with its trafficking transporter, LIMP2, 3) misfolded and unstable GCase is degraded by the proteasome, 4) failure of GCase to exit the Golgi, 5) GCase is inactive due to mutations at the active site, and 6) GCase activity is altered due to a Saposin C defect. (Fig. 4).

Regardless of degree of GCase deficiency, patients with *GBA1*-associated Parkinson disease appear to have increased α-synuclein aggregation. Post-mortem analysis of brain tissue from patients with Parkinson disease and those with Gaucher and Parkinson disease [50] demonstrated that decreases in GCase in the substantia nigra correlate with increases in α-synuclein levels. Moreover, Mazzulli et al. [51] showed that reduced GCase activity in cultured neurons resulted in reduced clearance, and subsequently increased levels, of α-synuclein protein. Decreases in GCase activity in the lysosome are also associated with accumulation of substrates GlcCer and GlcSph, with GlcSph being the more cytotoxic storage product [52]. GCase may also cleave galactosylceramide (GalCer) [53] to galactose and ceramide, thus loss of GCase activity may lead to GalCer accumulation as well. GCase has a broad enzymatic profile and may also act to transfer a glucose from GlcCer to cholesterol producing glycosylated cholesterol (GlcChol) [54]. Consequently, not only do GlcCer and GlcSph increase beyond homeostatic levels, but the accumulation of GlcChol and many other glucose-conjugated lipids may alter the cell’s ability to function. For example, it was recently noted in PD fibroblasts that the *GBA1* mutation N370S mediated lysosomal accumulation of cholesterol, that, in turn, may alter LIMP2 function [53]. In addition, accumulated GlcCer substrate was found to directly influence the conformation and solubility of α-synuclein by stabilizing the levels of soluble intermediates [51]. However, this association remains controversial because substrate accumulation is not observed in the brains of PD patients with heterozygous *GBA1* mutations [20]. It is possible that α-synuclein accumulation in lysosomes may reduce overall GCase activity in lysosomes, further compounding the issue. These findings underscore the complex cascade that can result from the loss of GCase and that may contribute to the generation of α-synuclein aggregates leading to Parkinson pathogenesis.

Loss of GCase activity can be acquired in many ways, but it is clear that a variety of factors including loss of GCase function, increased storage of intermediates like GlcCer, decreased transport of GCase from the ER or CMA disruption ultimately lead to increased α-synuclein accumulation, and a change from the soluble form to the aggregate form. The role of GCase in α-synuclein degradation appears to be important to maintaining homeostatic levels of monomeric α-synuclein in the cell, as enhanced GCase activity leads to reduced α-synuclein levels in iPSC-derived dopamineurgic neurons [55]. It has therefore been suggested that GCase and α-synuclein may have co-evolved to preserve a synergistic surface interaction around the GCase active site, but, if true, this role has yet to be defined [55]. Understanding the physical interactions between GCase and α-synuclein within the lysosomal pathway and the cascading effects on other aspects of Parkinson development may provide common intervention points for therapeutic approaches for both Gaucher and Parkinson disease.

### The protein structure of glucocerebrosidase and α-Synuclein and possible interacting domains

The mature GCase protein consists of 497 residues and has a calculated molecular mass ranging between 55 and 69 kDa depending on the number of occupied glycosylation sites. The X-ray crystal structure of GCase was first published in 2003 at a 2.0 Å resolution [56]. The protein consists of three domains. Domain I (residues 1–27 and 383–414) is composed of an antiparallel β sheet flanked by an amino terminal strand and a loop. This domain also contains two disulfide bridges (residues 4–16 and 18–23) which are required for correct folding of the protein [56]. Domain II (residues 30–75 and 431–497) is an immunoglobulin-like domain comprised of two closely associated β sheets. Domain III contains the catalytic domain (residues 76–381 and 416–430) and is a (β/α)_8_ triosephosphate isomerase (TIM) barrel with three free cysteines at residues 126, 248 and 342. Domains II and III appear to be linked by a flexible hinge, while domain I strongly interacts with domain III [56]. The common mutation N370S is located in the longest helix in the protein (helix 7) at the interface of domains II and III, but is too far from the active site to participate directly in catalysis. Several other mutations are found in this helix, all of which seem to point into the TIM barrel. Another common Gaucher mutation, L444P, is located in the hydrophobic core of the Ig-like domain (domain II). Any mutation in this domain may produce an unstable protein due to disruption of the hydrophobic core and altered folding of this domain [57].

There is also data suggesting that GCase can exist as a dimer in vivo*.* While different dimer forms are likely present, it was predicted that the form where the catalytic site is buried at the dimer interface is the preferred structure [58]. More recently, transition electron microscopy studies have clearly shown that GCase has a butterfly-shaped dimer structure both in solution and as a crystal, and that the dimer interface provided an allosteric binding pocket that may be significant for the design of future therapeutics [58].

In contrast to the highly structured GCase, α-synuclein is a small pre-synaptic protein of 140 amino acid with a less rigid structure and a propensity to form aggregates. It consists of three domains including the amino terminal lipid-binding α-helix, an amyloid-binding domain, and a carboxy-terminal acidic tail [59]. Using fluorescence and NMR spectroscopy, Yap et al. [60] showed that GCase interacts in close proximity with the C terminal of the α-synuclein protein in the acidic environment (pH 5.5) of the lysosome. It was postulated that α-synuclein is docked with GCase in the region of three highly conserved surface histidines (His-223, His-273 and His-328). The interacting C-terminus of α-synuclein (residues 126–140) are situated near loop 1 in the groove between the GCase C-terminus β sheet domain and the TIM barrel. It is suggested that interaction of α-synuclein with wildtype GCase promotes lysosomal degradation of α-synuclein or inhibits excessive α-synuclein accumulation. However, while evidence for the α-synuclein-GCase complex exists in vitro, not much is known regarding the mechanism through which the complex impacts α-synuclein stability or expression levels.

### ER stress, uncoupling of the protein response and autophagy

Misfolded proteins accumulating in the ER as a result of *GBA1* mutations can lead to ER stress while also activating the unfolded protein response (UPR). The UPR is upregulated in an attempt to protect the neural cell from the impact of chronic stress [61]. Two UPR chaperone mediators, GRP78 and calreticulin, were altered in *GBA1-*N370S dopaminergic neurons when compared to controls [62]. In an *A53T* mouse model of synucleinopathy [61], levels of the ER chaperone GRP78 were elevated in symptomatic mice [14]. It was also reported [63] that ER stress in a mouse model originated in the ER lumen/microsome fraction of the cell. In a *Drosophila* model containing a *GBA1* Rec*Nci*1 complex allele, the mutated GCase protein was also found to contribute to ER stress, resulting in alterations in eye development and increases in the ER stress marker, xbp1-EGFP [64]. Autophagosome markers (LC3 I and II, Beclin-1) were evaluated in iPSC-derived dopaminergic neurons from patients carrying a N370S allele, and independently in those from patients with a *SCNA* triplication. Both mutated lines showed disruption of the autophagy pathway [62] and up-regulation of the UPR [65]. The *SCNA* triplication demonstrated that α-synuclein accumulation significantly activates the UPR in a model independent of alterations in GCase activity. Consequently, cellular impairments that alter protein processing by a variety of mechanisms, including trapping of mutant GCase in the ER, can lead to α-synuclein accumulation and further disruption of vesicular trafficking. GlcCer accumulation in the lysosomes can also hinder lysosome-autophagy transport and degradation pathways and lead to increased α-synuclein aggregates [52, 66]. Conduritol–β-epoxide (CBE), an inhibitor of GCase, was also shown to increase α-synuclein accumulation in midbrain dopaminergic neurons [66], indicating that loss of GCase activity from mutant protein and/or increase in GlcCer are sufficient to promote α-synuclein aggregates. It is speculated that early intervention to alleviate ER stress before α-synuclein forms insoluble aggregates and fibrils may be a valuable therapeutic approach, since early aggregate forms can be reversed [67].

### Dysfunction of lysosomal trafficking

GCase reaches the lysosome by interacting with LIMP2, a protein that facilitates the trafficking of this acid hydrolase to the lysosomal lumen. Consequently, mutations in *SCARB2,* the gene that encodes LIMP2, can also contribute to reduced GCase activity [68]. Thus, impaired transport of GCase to the lysosome can contribute to reduced GCase activity even in the absence of GCase mutations. Other studies suggest that increases in α-synuclein disrupt ER to Golgi trafficking of GCase, setting up a bidirectional feedback loop, wherein decreases in GCase activity or increases in GlcCer yield increased levels of α-synuclein, that in turn accentuate α-synuclein aggregation. This is a GCase-specific defect, since leupeptin, a general lysosomal inhibitor, did not promote α-synuclein accumulation [68].

### Mitochondrial impairment/oxidative stress

There is evidence suggesting that mitochondrial import proteins may interact with α-synuclein via a cryptic mitochondrial import signal [69]. Mutations in *PARK2* (Parkin) and *PINK1* (PTEN-induced putative kinase), that result in monogenic Parkinson disease, are believed to impact mitochondrial function by increasing susceptibility to toxins [70]. Using a neuronopathic mouse model (K14-lnl/lnl) of Gaucher disease [71], Ossellame et al. [72] found that autophagic and proteasomal pathways were compromised in both neurons and astrocytes and showed insoluble α-synuclein accumulation in neurons. In this mouse, mitochondria were mis-shaped, fragmented and had reduced respiratory chain activity. In cell studies, reduction of GCase activity resulted in a progressive loss of mitochondria membrane potential required for ATP production, loss of respiratory complex activity, fragmented mitochondria and oxidative stress [73]. Finally, calcium regulation may also be affected in damaged mitochondria, yielding an altered membrane potential [74]. Mitochondrial dysfunction may also produce reactive oxygen species (ROS), causing chronic oxidative stress that may initiate misfolding of α-synuclein [75] and may initiate other degradative pathways in the neuron. Thus, secondary mitochondria dysfunction possibly results from a primary lysosomal defect (loss of GCase activity) that profoundly alters mitochondrial function. Cellular disruptions including ER stress, ROS and mitophagy may further compound the loss of cellular homeostasis and promote α-synuclein aggregation.

### Potential genetic modifiers of *GBA1* function

Genes that directly modulate the expression of another gene are known as genetic modifiers. While the association between *GBA1* and Parkinson disease is well-established, genetic modifiers may hold the key to elucidating *GBA1*-associated Parkinson genotype-phenotype correlation and underlying mechanisms of Parkinson pathogenesis. This subject has recently been extensively reviewed [76].

The search for modifiers began with a focus on candidate genes, but most of these pilot studies were not fruitful. Screening the closely located *MTX1* gene in 600 Ashkenazi Jewish patients with Parkinson and 353 control patients suggested that homozygosity for the *MTX1* c.184 T > A (p.S63 T) alteration induces earlier onset of Parkinson disease in affected patients [77]. A GWAS performed in 153 Ashkenazi Jewish patients with *GBA1*-associated Parkinson disease highlighted the gene *BIN1* as a potential candidate modifier gene for early-onset *GBA1*-associated Parkinson disease *BIN1* encodes the Bridging Integrator 1 (BIN1) protein, a protein involved in CNS synaptic vesicle endocytosis [78]. Mutations in *BIN1* are thought to induce early-onset Parkinson disease in patients with at least one mutated *GBA1* allele. However, this finding did not reach genome-wide significance. A different GWAS identified *TMEM175* (transmembrane protein 175), a lysosomal K+ channel, as a potential modifier gene in patients with Parkinson disease. Considering its role in modulating lysosomal pH, mutations in *TMEM175* are thought to further impair GCase activity as well as increase exogenous α-synuclein levels [79]. Larger patient studies may aid in the identification of further relevant genetic modifiers. This can be accomplished by performing whole exome or genome sequencing of cohorts with Parkinson disease with and without *GBA1* mutations to see if those with *GBA1* mutations share other specific variants. Likewise, genomic sequencing of cohorts of older patients with Gaucher disease with and without parkinsonism may prove fruitful.

Additionally, it remains unclear whether known Gaucher modifiers like prosaposin (*PSAP)* or LIMP2 (*SCARB2)* also play a role in patients with *GBA1*-associated parkinsonism. Rothaug et al. [80] have shown that mice featuring a double knockout of the lysosomal targeting gene *SCARB2* exhibit elevated levels of GluCer, α-synuclein accumulation and dopaminergic neurodegeneration. However, further clinical studies are required to confirm this putative connection in human subjects. While recent reviews have extensively summarized the role of various lysosomal genes and mutations involved in Parkinson pathogenesis identified through methods such as GWAS and family studies (including *SCARB2, LRRK2, SMPD1, PARK2, PINK1, PARK7*, and others), apart from *SCARB2*, the potential interactions between these genes and *GBA1* on overall lysosomal function remain relatively uncharacterized [81–83].

Finally, there is a strong possibility that epigenetics plays a role in susceptibility to Parkinson disease by modulating the *GBA1* gene. Epigenetics, known as the post-transcriptional modification of genetic expression, has been implicated in complex neurological disorders such as Alzheimer disease via histone acetylation of *BACE1*, or schizophrenia via hypomethylation of *COMT* [84, 85]. While no published epigenetic studies of Gaucher-associated Parkinson disease exist to date, they may provide insight as to how siblings with the same *GBA1* genotype may develop discordant parkinsonian phenotypes [86, 87].

### Modelling Gaucher disease in vivo and in vitro

Many approaches are utilized to study the pathological alterations driven by deficient GCase. Yet, the relationship between Gaucher mutations and the increased risk for Parkinson disease remains unclear. Many of the animal and non-animal models developed to study Gaucher-associated Parkinson disease (GD-PD) pathology have contributed significant information about different aspects of these diseases.

Researchers have turned to vertebrate and non-vertebrate models of *GBA1*-associated parkinsonism to address selected, unresolved topics, such as the specific role of the GCase pathway in Parkinson pathogenesis and to test novel treatments for Gaucher disease. While animal models have inherent limitations, they remain useful, for the animal’s environment and genetics can be manipulated while still partially recapitulating the complex neural system of humans complex neural system. However, these diverse models fail to replicate the human brain’s complex cognitive and motor interconnections. Other promising models that that offer a way to elucidate possible pathogenic mechanisms are neurologic models derived from patient cells using the pluripotent reprogramming approaches developed by Yamanaka et al. [88]. Overall, current GD-PD models often face an issue of prioritization between achieving desired Parkinson-like phenotypes or maintaining a realistic *gba* genotype. Modeling susceptibility to Parkinson disease, let alone Gaucher-associated Parkinson disease, remains exceedingly difficult for these reasons.

### *GBA1*-associated Parkinson models in diverse non-vertebrate organisms

Non-vertebrate organisms such as *Caenorhabditis elegans*, (worms) *Drosophila melanogaster* (fruitfly*)* and the vertebrate fish, *Oryzias latipes* (medaka) have been used to demonstrate the relationship between Gaucher disease and Parkinson disease. One remarkable advantage of these models over mouse models is that animals homozygous for the null *gba* allele remain viable, rather than exhibiting a neonatal lethal phenotype [89]. *C. elegans* are used to evaluate movement disorders because of their simple neurological system, transparent body and easily observable and stereotyped motor behavior [90]. *C. elegans* with depleted GCase activity do exhibit higher α-synuclein levels than their wildtype counterparts [51], resembling what is observed in human cellular models and mouse models. *GBA1* orthologs in *D. melanogaster* located on chromosome 3 (*dGBA1a* and *dGBA1b*), encoding proteins with ~ 31% and ~ 49% homology to human GCase, respectively, provide enough similarity to create a Gaucher-like condition when altered [91]. Mutations in either of these orthologs creates a truncated protein; compound heterozygous flies representing the human *GBA1* carrier status demonstrate an elevated UPR and decreased survival. Additionally, knock-in fly models expressing the human mutations N370S and L444P have been used to successfully characterize UPR activation and locomotor defects in the presence of mutated *GBA1* mRNA [91]. Overexpression of the human transgene *SNCA*^*A53T*^ in animal models is a commonly used approach to exacerbate Parkinson disease progression in fly, mouse, and even macaque models. Knock-out flies deficient in *gba*, crossed with those carrying a *SNCA*^A53T^ transgene displayed increased α-synuclein aggregation, loss of dopaminergic neurons, negative geotaxis and eye defects [92, 93]. Knockouts of *gba* in medaka, a fish model, show a neuronopathic Gaucher disease-like pathology, along with elevated α-synuclein levels and abnormal swimming movement [94]. Research utilizing these models has contributed valuable information regarding the role of the ubiquitin protease system and α-synuclein in Parkinson pathogenesis and seem to confirm, species wide, an evolutionarily conserved relationship between GCase and α-synuclein.

### Genetically or chemically modified mouse models of *GBA1*-associated Parkinson

A great deal of work has gone into characterizing mouse models of GD-PD, created by knocking out or knocking down GCase activity. GCase impairment in mouse models is accomplished in two ways: genetic or chemical [95].

While genetic mouse models remain the more popular of the two methods, different shortcomings prevent any one model from exhibiting an accurate *GBA1*-associated parkinsonian phenotype. Due to the wide range in *GBA1* genotypes encountered in patients with Parkinson disease, researchers have attempted to introduce a battery of *gba* genotypes to model GD*-*PD, primarily based upon commonality, pathogenicity, and known neurological manifestations associated with human mutations. Both heterozygous and homozygous mutant models have been used to emulate *GBA1* carriers and patients with Gaucher disease, respectively. Examples of heterozygous GD*-*PD mice include the L444P/+, KO/+, and D409H/+ models, amongst others [96–98]. Unfortunately, these “carrier” mice do not exhibit Gaucher or Parkinson-like phenotypes, and so more severe, homozygous models are used instead. These models have displayed pathological alterations reminiscent of Parkinson disease, such as α-synuclein accumulation, dopaminergic neurodegeneration and motor impairment. Another approach to model GD-PD is to cross *gba* mutation-carrying mice with an established model of parkinsonism, such as mice overexpressing the human transgene *SNCA*^*A53T*^ [96, 98]. In addition to impaired GCase, these compound mutant mice exhibit an accelerated rate of α-synuclein accumulation, and an accelerated Parkinson-like phenotype. An example is crosses between the *gba1* D409H/D409H mouse and the *SNCA*^A53T^ mouse. The double mutant mice display substantia nigra-specific neurodegeneration, an increase in α-synuclein levels, and impaired memory and motor behavior [52]. Another murine example, generated by administrating the substantia nigra-specific neurotoxin MPTP (1-methyl-4-phenyl-1,2,3,6-tetrahydropyridine) to L444P/+ mice, demonstrate more severe Parkinson-like pathology than mice created by either mechanism alone [96, 99].

Chemically inducing a Gaucher-like phenotype through the administration of CBE, a direct GCase competitive inhibitor, is also employed to model Gaucher-like pathology [89]. High doses of CBE, thought to model neuronopathic Gaucher disease, induce increased monomeric α-synuclein levels [100]. Lower CBE doses over a sustained period of time more closely resemble *GBA1* mutation carriers, with approximately 50% residual GCase activity, and creates a more Parkinson-like phenotype, with increased oligomeric α-synuclein levels, brain-wide neurodegeneration, and microglial activation [101]. CBE models have major limitations, as the inhibitor can impact upstream pathways and non-specific chemical targeting, but they remain useful in certain circumstances.

### Development of human iPSC models of *GBA1*-associated parkinsonism

The brain may be the most complex organ in the body, and its complexity limits access to neurospecific cell types for study. Yamanaka et al. [88] introduced the ability to reprogram somatic cells into induced pluripotent stem cells (iPSC) with the capability to be differentiated into a variety of somatic cell types, including neural sub-types. The use of iPSC technology to study brain specific cell subtypes in a patient context has advanced our understanding of function and the uniqueness of the cellular interactions in many neurodegenerative diseases (reviewed in [102, 103]. Patient and control iPSC lines to can be used to generate neural-specific cells [104, 105] either directly using chemical signals (SMAD) to produce iNeurons or indirectly through embryoid bodies that develop into early neuroepithelial progenitor cells [106]. The iPSC approach to model human neurodegenerative diseases also provides a scalable system that can by-pass the limitation of availability of patient biopsy material, instead using readily available patient fibroblasts or blood cells to produce iPSCs. Disease can then be investigated in the genetic context of the patient’s cells, allowing exploration of both pathology and therapeutics. There are now many examples of iPSC models to probe neurodegenerative diseases [107] including Gaucher disease [108, 109] Parkinson disease [65, 110] and Parkinson disease with *GBA1* heterozygosity [111–113].

The iPSCs can be further differentiated to neural subtypes such as dopaminergic (DA) neurons, cortical neurons and radial glial cells [114]. While it has been difficult to study progressive, adult-onset diseases because of limited access to brain tissue and/or specific cell populations, many investigators now use iPSC methodologies to tackle the intricacies of neurodegenerative disease. One focus of iPSC studies in Gaucher disease is to evaluate lysosomal trafficking and autophagy impairment [115] and to reproduce pathologic hallmarks [116]. In parkinsonism, investigators examined cortical and dopaminergic neurons as well as astrocytes derived from patient iPSCs. One such study revealed defective synaptic connectivity in a familial Parkinson model [110]. Other iPSC models of parkinsonism have shown altered autophagic flow in DA neurons [117], alterations of vesicle trafficking in neural progenitor cells [118], impaired tau expression and alterations of mitochondrial function [119] in cortical neurons and, finally, increased susceptibility to oxidative stress in astrocytes [120]. In patient-specific iPSCs containing the *LRRK2* G2019S mutation, Domenico et al. (2019) reported dysfunctional chaperone-mediated autophagy and progressive accumulation of α- synuclein in iPSC-derived astrocytes [121]. Patient-derived iPSC disease models are now an evolving resource that may contribute unique insights into understanding progressive, adult-onset neurodegenerative diseases [122] like parkinsonism and lysosomal storage disorders. Another recent in vitro research approach to investigate human brain function is brain organoids [123–125]. These three-dimensional models rely on the pluripotent iPSCs to self-organize in vitro to neurological tissue structures. While organoids to date are largely representative of early fetal development, they have already shown utility in assessing microcephaly resulting from Zika virus expression [126, 127]. As organoid protocols are refined and validated, genomic engineering approaches will be applied to generate organoids that will permit specific mutations to be evaluated in these more complex, neurospecific models [128].

### Prospects for treatment of *GBA1*-associated parkinsonism

As discussed above, current treatments for Gaucher disease are ERT and SRT, both FDA-approved interventions designed to generate and maintain a more normal GCase-substrate ratio in patients. While these treatments have substantially improved the visceral symptoms of Gaucher disease, current forms of ERT fail to cross the blood-brain barrier, and as a result, do not prevent or reverse the neuronopathic manifestations of Gaucher disease [4]. Considering the heavily implicated role of GCase in Parkinson pathogenesis, developing an effective treatment that can restore neural GCase levels could not only drastically improve the quality of life for patients with neuronopathic Gaucher disease, but could also potentially prevent Parkinson onset in patients susceptible to Gaucher-associated Parkinson disease or even idiopathic parkinsonism. Currently brain penetrant forms of SRT are in clinical trials for patients with Parkinson disease who are heterozygous carriers of *GBA1* mutations. However, there is not solid evidence that there is substrate accumulation in the brains of *GBA1* mutation carriers, and homozygotes are not included in the study. Currently, three types of novel treatments are being investigated in addition to ERT and SRT (Fig. 4).

### Gene therapy

One possible mechanism to overcome the blood-brain barrier is the direct delivery of corrected genetic material to the affected tissue, otherwise known as gene therapy. While a variety of viral vectors are available, the most common type used in relation to *GBA1* is adeno-associated viral infection (AAV). AAV owes its popularity due to its ability to reliably deliver corrected DNA into chromosome 19 of the cell through non-pathogenic infection with nearly perfect specificity, reducing the need to implant excessive copies of the correctional gene into the cell [129, 130]. AAV-mediated delivery of *GBA1*, otherwise known as AAV-*GBA1*, has been tested in animal models to assess its effect on Gaucher and/or Parkinson biomarker levels [131, 132]. Regardless of the *gba* genotype, murine brain hemispheres injected with AAV-*GBA1* demonstrated improved GCase activity, decreased GluCer and GluSph and lowering of α-synuclein levels, and exhibited decreased neurodegeneration and neuroinflammation, compared to the complementary brain hemisphere injected with vehicle only [99, 132–134]. Massaro et al. [132], using WT, KO/+, and conditional KO/KO mice, also demonstrated that early therapeutic intervention via AAV9-GBA1 drastically improved the mouse lifespan and GCase activity and decreased GluCer substrate levels. While AAV9-GBA1 did not completely rescue the mouse’s phenotype, it clearly ameliorated many of the murine symptoms. Ultrasound-guided in *utero* delivery in fetal mice and macaque brains similarly stabilized symptoms such as motor coordination and long-term microglial and astrocyte activation [132]. While this method carries great potential as a single-dose, long-term solution to neuronopathic Gaucher disease, in mice it was found to work best with in *utero* or neonatal administration, as neurons subsequently have limited regenerative capacities [132].

### Therapy with pharmacological chaperones

Chemical chaperones, small molecules that stabilize and refold misfolded proteins, are small enough to effectively cross the blood-brain barrier, making them a promising candidate for therapeutic research. Pharmacological chaperones may be able to stabilize misfolded GCase in the ER, knock down initiation of UPR and ER stress responses, and thus suppress apoptosis and mitochondria dysfunction [135]. A GCase chaperone may also facilitate post-ER trafficking to the lysosome, enhancing delivery of mutated GCase protein to the lysosome where there may still be adequate GCase activity [135]. Molecular chaperones are separated into two broad categories depending on their mechanism: extrinsic and intrinsic. Small molecule chaperones of GCase can be further separated into chaperones that bind to the active site as competitive inhibitors, or non-inhibitory chaperones that primarily enhance enzymatic activity.

Extrinsic chaperone proteins serve to stabilize and refold proteins during periods of extreme stress that disrupt proteostasis, such as heat shock, cold, UV light, hypoxia, or wound healing. Members of the heat shock protein 70 (Hsp70) family are essential for proper GCase and lysosomal function and are known to work with co-chaperones such as TCP1 to identify and refold mutated GCase [136]. Evidence suggests that administrating chemical chaperones to elevate endogenous chaperone protein levels may provide the key to refolding GCase and restoring normal enzymatic activities in the brain. Arimoclomol, one such chemical compound, induces the heat shock response, thereby amplifying Hsp70 and other heat shock proteins. Administering arimoclomol to fibroblasts derived from patients with genotype L444P/L444P improved GCase activity at a rate similar to approximately one unit of the standard ERT drug, alglucerase [136]. A similar molecular chaperone, celastrol, acts by stabilizing the BAG protein family regulator 3 (BAG3) to refold mutant GCase [85].

Intrinsic chaperone proteins are chemical compounds that serve to directly stabilize misfolded GCase in the ER, allowing more functional proteins to form that can evade the ER-associated degradation pathway. Ideally, these small molecules selectively bind to mutant GCase at the neutral pH of the cytosol, and then lose their binding affinity as the enzyme enters the acidic pH of the lysosome. Ambroxol, one such chemical compound, was selected as a candidate chaperone protein identified from high-throughput screening of an FDA-approved chemical library composed of 1040 compounds [137]. Administering ambroxol to patient-derived mutant *GBA1* cell lines rescued GCase activity and increased GCase levels on a dose-dependent basis [138]. While murine Gaucher models have responded favorably to ambroxol administration, Parkinson related symptomology does not seem to be affected [138]. L444P/+ mice treated with oral ambroxol for 12 days exhibited increased GCase levels compared to vehicle controls, but there was no change in α-synuclein levels [139]. NCGC607, a small-molecule noninhibitory chaperone of GCase, is another chaperone protein identified by high-throughput screening. Administration of NCGC607 to iPSC-derived dopaminergic neurons from patients with both Gaucher disease and Parkinson disease showed that the chaperone protein was able to reduce substrate accumulation and improve GCase activity levels, highlighting its potential as an effective therapeutic. Interestingly, NCGC607 administration was also able to decrease α-synuclein levels in the neurons derived from patients with parkinsonism, suggesting this compound’s potential utility as a treatment for parkinsonism [140]. Mazzulli et al. [141] reported that increasing GCase activity by administration of a different non-inhibitory small molecule chaperone, NCGC758, resulted in α-synuclein clearance regardless of *GBA1* mutation status. Lastly, isofagomine (IFG) is an aza-sugar has also been evaluated as a chaperone protein, binding to both the wild-type and mutant versions of GCase. Cell lines from patients with missense *GBA1* mutations showed markedly improved GCase levels after incubation with IFG. While no mouse study has been conducted to investigate the impact of IFG on parkinsonian manifestations, transgenic mouse models homozygous for missense *gba* mutations have shown an improvement in overall organ size and GCase activity after IFG treatment [99, 142, 143]. Thus, from preliminary data, pharmacological chaperones represent a potential therapeutic approach for altering GCase activity by assisting in clearing the cell of early α-synuclein accumulation.

Overall, pharmacological chaperone therapy presents some advantages over the current standard of care treatments for Gaucher disease, ERT and SRT. In addition to potentially being able to cross the blood-brain barrier, small molecular compounds are less expensive to manufacture, and can be taken orally instead of through intravenous infusions. From preliminary data, pharmacological chaperones could represent a potential therapeutic approach for preventing early α-synuclein accumulation. However, chaperone proteins only stabilize and improve the performance of misfolded GCase protein; thus, its use may be limited in the case of null mutations such as c.84insG. Furthermore, chaperone proteins have difficulty refolding L444P mutated GCase, because the altered amino acid lies outside the catalytic domain of the protein, in the immunoglobin domain [135, 144]. Administering a chaperone that directly binds to this non-catalytic domain could rectify this issue, but brings into question whether specific chaperone proteins are required for each domain of GCase, and by extension each *GBA1* genotype. However, the potential benefits that chaperone therapy provides for patients with a missense *GBA1* mutation (representing the majority of patients) deserve consideration.

### Histone deacetylase inhibitors

Another class of small molecules that may serve to stabilize mutant GCase are histone deacetylase inhibitors (HIDACis), known for their effect on proteostasis [145]. Histone deacetylase proteins (HDACs) operate through post-transcriptional modification of histones, transcriptional modifiers, and chaperone proteins. These processes are significantly upregulated in patients with Gaucher disease [146]. Specifically, HIDACs have been shown to remove acetyl moieties from Hsp70, Hsp90, and tubulin, thereby enhancing their activity [147]. Heat shock proteins such as Hsp70 and Hsp90 display remarkably diverse functions, where in addition to stabilizing misfolded proteins, they also stabilize proteasome complexes to direct UPR-related degradation. Inhibiting this process through HIDACis inhibits Hsp90’s ability to direct protein degradation, preventing recognition and degradation of mutant GCase, thereby increasing its quantity and catalytic activity in fibroblasts cell lines [145, 148]. The administration of two HDACis, suberoylanilide hydroxamic acid (vorinostat) and LB-205, ameliorated the performance of fibroblasts derived from patients with Gaucher disease, along with other lysosomal storage disorders such as Niemann-Pick Type C disease [145, 148–150].

## Conclusions

The link between *GBA1* and parkinsonism was surprising, and only recognized because of clinical findings that led to this association between a rare and common disorder. Despite a wealth of knowledge concerning the structure and function of glucocerebrosidase, our understanding of the role of this enzyme in Parkinson pathogenesis remains incomplete. Clearly there is an inverse relationship between levels of glucocerebrosidase and α-synuclein, suggesting that therapeutics aimed at enhancing glucocerebrosidase levels may have utility in the treatment of Parkinson disease. These are exciting times for those in the Gaucher field, because the increased attention focusing on glucocerebrosidase is also likely to yield new therapies for patients with Gaucher disease. Further exploration into potential genetic modifiers and epigenetic modifications will likely enhance our understanding of the role of this lysosomal protein in the etiology of Parkinson disease.

## Data Availability

Not applicable.

## Abbreviations

AAV: Adeno-associated virus
BAG3: BAG protein family regulator 3
BIN1: Bridging integrator 1
CBE: Conduritol–β-epoxide
CMA: Chaperone mediated autophagy
ER: Endoplasmic reticulum
ERT: Enzyme Replacement Therapy
GalCer: Galactosylceramide
*GBA1*: Glucosylceramidase Beta 1
GCase: Glucocerebrosidase
GD-PD: Gaucher-Parkinson
GlcCer: Glucosylceramide
GlcChol: Glycosylated Cholesterol
GlcSph: Glucosylsphingosine
HDAC: Histone deacetylase
HDACi: Histone deacetylase inhibitor
Hsp: Heat shock protein
IFG: Isofagamine
iPSC: induced pluripotent stem cells
LAMP2a: Lysosome-associated membrane protein 2a
*LIMP2*/SCARB2: Lysosomal Integrated Membrane Protein 2/Scavenger Receptor Class B Member 2
MPTP: 1-methyl-4-phenyl-1,2,3,6-tetrahydropyridine
*MTX1*: Metaxin1
PARK2: E3 ubiquitin-protein ligase parkin
PINK1: PTEN-induced kinase 1
PSAP: Prosaposin
ROS: reactive oxygen species
*SCNA*: Alpha-synuclein gene
SRT: Substrate Reduction Therapy
TFEB: Transcription Factor EB
TIM: Triosephosphate isomerase
TMEM175: Transmembrane protein 175
TPS3: Thrombospondin 3
UPR: Unfolded protein response
